# Integrin-mediated osteoblastic adhesion on a porous manganese-incorporated TiO_2_ coating prepared by plasma electrolytic oxidation

**DOI:** 10.3892/etm.2013.1204

**Published:** 2013-07-04

**Authors:** ZHENXIANG ZHANG, BEIBEI GU, WEI ZHU, LIXIAN ZHU

**Affiliations:** Orthopedic Department, The Affiliated Taizhou People’s Hospital of Nantong University, Taizhou, Jiangsu 225300, P.R. China

**Keywords:** plasma electrolytic oxidation, manganese, TiO_2_, MG63, adhesion

## Abstract

This study was conducted to evaluate the bioactivity of manganese-incorporated TiO_2_ (Mn-TiO_2_) coating prepared on titanium (Ti) plate by plasma electrolytic oxidation (PEO) technique in Ca-, P- and Mn-containing electrolytes. The surface topography, phase and element compositions of the coatings were investigated using scanning electron microscopy (SEM), X-ray diffraction (XRD) and energy dispersive spectrometry (EDS), respectively. The adhesion of osteoblast-like MG63 cells onto Ti, TiO_2_ and Mn-TiO_2_ surfaces was evaluated, and the signal transduction pathway involved was confirmed by the sequential expression of the genes for integrins β_1_, β_3_, α_1_ and α_3_, focal adhesion kinase (FAK), and the extracellular regulated kinases (ERKs), including ERK1 and ERK2. The results obtained indicated that Mn was successfully incorporated into the porous nanostructured TiO_2_ coating, and did not alter the surface topography or the phase composition of the coating. The adhesion of the MG63 cells onto the Mn-incorporated TiO_2_ coating was significantly enhanced compared with that on the Mn-free TiO_2_ coating and the pure Ti plates. In addition, the enhanced cell adhesion on the Mn-TiO_2_ coatings may have been mediated by the binding of the integrin subunits, β_1_ and α_1_, and the subsequent signal transduction pathway, involving FAK and ERK2. The study indicated that the novel Mn-TiO_2_ coating has potential for orthopedic implant applications, and that further investigations are required.

## Introduction

Titanium (Ti) and its alloys are frequently used as orthopedic implant materials, due to their mechanical strength, chemical stability and biocompatibility (1,2). It has been demonstrated that the biocompatibility of Ti is closely related to the properties of the surface oxide layer (predominantly titanium dioxide, TiO_2_), with regard to its structure, morphology and composition. However, TiO_2_ does not exhibit sufficient bioactivity to form a direct bond with the juxtaposed bone, and this may translate into a lack of osseointegration, leading to the long-term failure of the implant (3–5). Combining the TiO_2_ with bioactive materials is recognized to be an effective method of overcoming this drawback.

There has recently been an increased focus on the effects of trace elements on biological processes, particularly in the field of bone formation and in the study of essential elements. With regard to the divalent cations, there have, to date, been a variety of studies on manganese (Mn) (6–9). Mn is an essential trace element in the human body, and it has the most potent capacity for binding to integrins, and for mediating the binding of ligands to various integrins, at low concentrations (6,10–12). Previous studies have endeavored to use Mn to enhance the osteoconductivity of a bioinert Ti substrate, by means of its potent cell adhesion-promoting effect (7,9,13,14). However, there have not been any studies into Mn-containing TiO_2_ ceramics or coatings, and the effects of Mn on the composition, microstructure and biological responses of TiO_2_ have not been elucidated. Moreover, the signal transduction pathway that mediates the effects of Mn on osteoblastic adhesion has not been studied. Therefore, the aim of the present study was to investigate the preparation and characterization of Mn-containing TiO_2_ coatings.

Plasma electrolytic oxidation (PEO), also known as microarc oxidation, is a relatively convenient and effective technique for the preparation of TiO_2_-based coatings on a Ti substrate (15–17). PEO coatings are, in general, porous and nanostructured, and this has been demonstrated to be beneficial to osteoblast adhesion and proliferation (15,17). At present, the PEO process is widely applied to the biofunctionalization of titanium, in order to create bioactive porous oxide coatings (18). In addition, many biological elements, for example calcium (Ca) and phosphorus (P), may be effectively incorporated into the PEO-evoked TiO_2_ coating through the supplementation of the electrolyte (19,20). In the present study, a porous Mn-TiO_2_ coating was prepared by PEO in an electrolyte containing Ca, P and Mn. In addition, the adhesion behavior of osteoblast-like MG63 cells onto the Mn-TiO_2_ coating, and the corresponding signal transduction pathway, were investigated.

## Materials and methods

### Preparation of samples

The study used commercially pure Ti plates with dimensions of 10×10×1 mm. The Ti was mechanically polished using silicon carbide (SiC) abrasive sandpaper. For the TiO_2_ coatings, the Ti plates were anodized in an electrolyte containing 0.05 mol/l glycerophosphate disodium salt pentahydrate (C_3_H_7_Na_2_O_6_P.5H_2_O, GP) and 0.1 mol/l calcium acetate monohydrate [(CH_3_COO)_2_Ca.H_2_O, CA], while for the Mn-TiO_2_ coatings, 0.04 mol/l manganese acetate [Mn(CH_3_COO)_2_·2H_2_O] was added into the electrolyte. The current density, frequency, duty cycle and duration time were fixed at 16.5 A/dm^2^, 800 Hz, 10% and 4 min, respectively. Following the PEO treatment, the samples were washed with deionized water, and dried in air.

The surface characterization of the PEO-treated samples was performed using scanning electron microscopy (SEM), with a S-4200 scanning electron microscope (Hitachi, Tokyo, Japan), X-ray diffraction (XRD), with a D/MAX-2550 diffractometer (Rigaku Corporation, Tokyo, Japan) and energy-dispersive X-ray spectrometry (EDS) attached to an electron probe X-ray microanalysis system (EPMA), using an XA-8100 microprobe (Hitachi, Tokyo, Japan). The surface roughness (Ra) of the samples was measured using a surface profiler (Hommel Tester T8000; Hommelwerke GmbH, Villingen-Schwennigen, Germany) with a scan distance of 4.8 mm and a scan rate of 0.5 mm/sec. The scan was performed on each sample three times at different locations on the sample.

### Cell culture

The osteoblastic MG63 cells were cultured in α-minimum essential medium (MEM), supplemented with 100 mg/ml penicillin G, 50 mg/ml gentamicin, 3 mg/ml amphotericin B and 15% newborn bovine serum, at 37°C in a humidified atmosphere of 95% air and 5% CO_2_. The osteoblasts were trypsinized (0.25% trypsin, 0.1% glucose, citrate-saline buffer; pH 7.8) prior to confluent growth, in order to avoid post-confluence differentiation effects, counted electronically with a cell counter (Coulter Electronics Ltd., Luton, UK) and plated onto the test and control surfaces at a density of 30,000–100,000 cells/ml. A total of 31.5 mg/ml sodium-β-glycerol phosphate and 0.58 mg/ml L-ascorbic acid phosphate magnesium salt n-hydrate were added to the supplemented α-MEM over the course of the experiments. Cells were not used above passage number 20.

### MTT assay

An MTT assay was used to determine the cell attachment. The cells were seeded at a concentration of 2×10^5^ cells/cm^2^ onto the disks of the Ti plates, and the TiO_2_ and Mn-TiO_2_ coatings, and were cultured on the disks for 1, 8, 16 and 24 h, respectively, in a 37°C incubator with 5% CO_2_. At the pre-determined time points, each disk was transferred to a well in a new 24-well plate, and 1.5 ml medium was added to each disk. A total of 150 μl freshly prepared 5 mg/ml MTT was added to each well where a disk was present. The plates were then placed in an incubator at 37°C for 3 h, prior to the supernatant of each well being removed, and replaced with acidified isopropanol (0.04 M HCl in isopropanol). This was subsequently mixed thoroughly to dissolve the dark-blue crystals. The absorbance was measured with a spectrophotometer at a wavelength of 570 nm, with a subtraction of the absorbance at 650 nm. The cell number was determined using a linear correlation between the absorbance and MG63 cell concentration.

### Cell morphology

The cells were seeded onto the Ti plates and the TiO_2_ and Mn-TiO_2_ coatings at a density of 1×10^4^/cm^2^ in α-MEM supplemented with 10% phosphate-buffered saline (PBS), and were cultured under standard cell culture conditions for 16 h. The specimens were then separated from the medium, washed twice in PBS and fixed with 2.5% glutaraldehyde in PBS for 1 h at room temperature. Following this, the samples were dehydrated in a graded ethanol series of 50, 70, 80 and 95% ethanol for 5 min, as well as twice with 100% ethanol for 15 min. Subsequently, the samples were stored overnight in a thermo-ventilated oven at 37°C, prior to undergoing gold metallization using Emscope SC500 apparatus (Quorum Technologies Ltd., Ashford, UK), and SEM analysis with a Philips XL30 FEG scanning electron microscope (Philips, Amsterdam, the Netherlands).

### Quantitative polymerase chain reaction (qPCR) analysis

The gene expression of the integrin subunits β_1_, β_3_, α_1_ and α_3_, as well as FAK, ERK1 and ERK2, were determined using qPCR analysis. The cells were seeded onto Mn-TiO_2_ and TiO_2_ coatings, in addition to Ti plates, in 24-well plates at a density of 2×10^4^ cells/well, and cultured for 1, 8, 16 and 24 h, respectively. The total cellular RNA was extracted using TRIzol^®^ reagent (Invitrogen Life Technologies, Carlsbad, CA, USA), in accordance with the manufacturer’s instructions. To obtain first-strand cDNAs, 1 μg total RNA extract was used for reverse transcription. The reactions were performed using a RevertAid™ First Strand cDNA Synthesis kit (Thermo Fisher Scientific, Inc., Waltham, MA, USA), in a final volume of 20 μl, at 42°C for 60 min, and were then terminated by heating at 70°C for 5 min. Glyceraldehyde-3-phosphate dehydrogenase (GAPDH) was used as the housekeeping gene. The primer sequences utilized are displayed in Table I.

The amplification process was performed using a Maxima SYBR-Green qPCR Master Mix in an Applied Biosystems 7500 RT-PCR system (Applied Biosystems, Foster City, CA, USA). The reaction volume was 25 μl, containing 12.5 μl SYBR-Green qPCR Master Mix, 1 μl each primer (0.3 μM), 2 μl template DNA and 8.5 μl nuclease-free water. The initial denaturation was carried out at 95°C for 600 sec, with denaturation at 95°C for 15 sec, annealing at 60°C for 30 sec and then 40 cycles of extension at 72°C for 30 sec. At the end of the PCR cycles, a melting curve analysis was performed to determine the specificity of the PCR product. The mRNA content of each gene was normalized to the quantity of GAPDH mRNA, and the average threshold cycle (CT) values were used to quantify the gene expression in each sample: ΔCT = ΔCT(experiment)-ΔCT(control). The relative gene expression (fold change) was obtained by transforming the logarithmic values into absolute values using 2^−ΔΔct^.

### Statistical analysis

One way analysis of variance (ANOVA) and Tukey’s multiple comparison tests were performed to detect any significant effects that occurred as a result of the experimental variables. The results were analyzed using the Student’s t-test, with a sample number including ≥4 samples. The error bars represent mean ± standard deviation. P<0.05 was considered to indicate a statistically significant difference.

## Results

**Figure 1:**
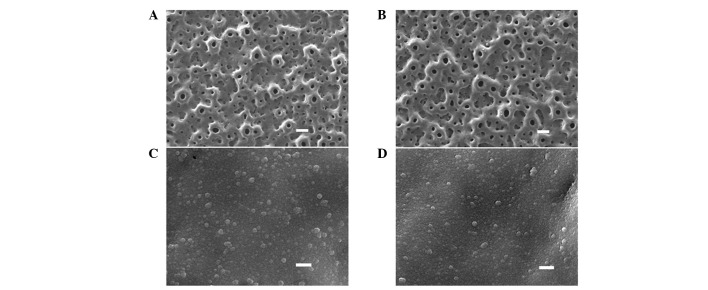
Surface morphologies of the (A and C) titanium dioxide (TiO_2_) and (B and D) manganese (Mn)-incorporated-TiO_2_ coatings at different magnifications (A and B; magnification, ×1,000; bar=5 μm; C and D; magnification, ×50,000; bar=100 nm).

**Figure 2:**
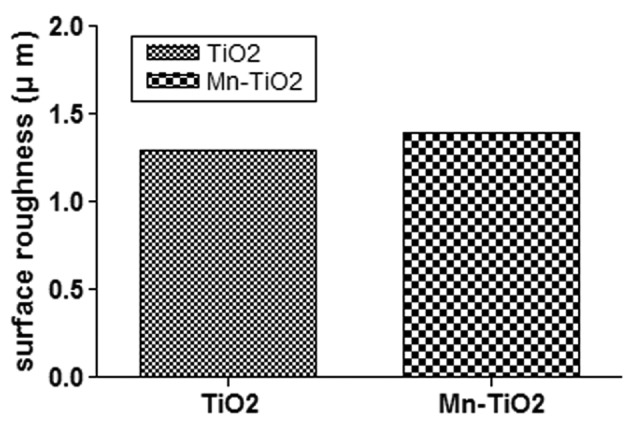
Surface roughness of the titanium dioxide (TiO_2_) and manganese (Mn)-incorporated-TiO_2_ coatings.

As demonstrated in Fig. 1, the low magnification views (x1,000) revealed that the TiO_2_ (Fig. 1A) and Mn-TiO_2_ (Fig. 1B) coatings were porous, with a pore size of <5 μm. The pores were well separated, and homogeneously distributed over the coating surfaces. The high magnification views (x50,000) indicated that the TiO_2_ (Fig. 1C) and Mn-TiO_2_ (Fig. 1D) coatings were covered by nanograins of ~30–50 nm. No obvious differences in morphology were observed between the TiO_2_ and Mn-TiO_2_ coatings. Fig. 2 indicates that there was no significant difference between the Ra of the TiO_2_ and Mn-TiO_2_ coatings (1.3±0.1 μm and 1.4±0.2 μm, respectively).

**Figure 3:**
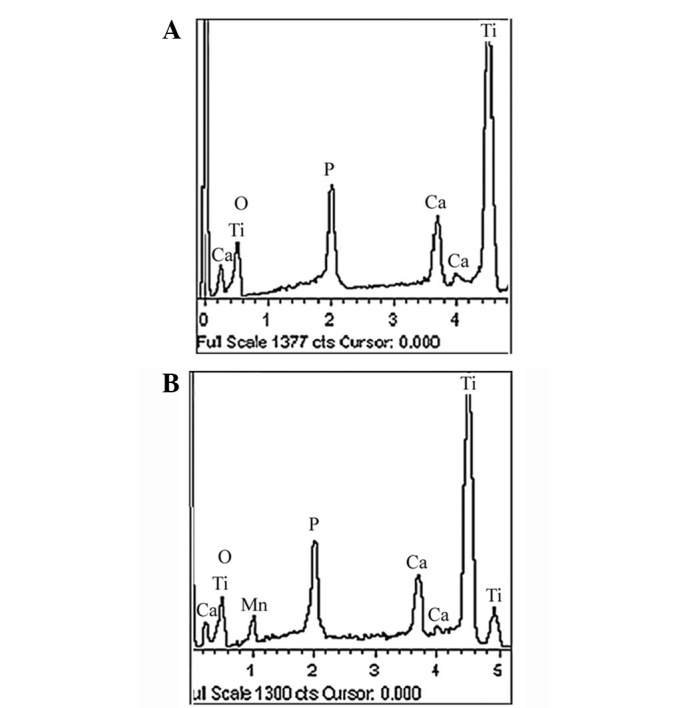
Energy dispersive spectrometry (EDS) spectra of the (A) titanium dioxide (TiO_2_) and (B) manganese (Mn)-incorporated-TiO_2_ coatings. Ca, calcium; Ti, titanium; O, oxygen; P, phosphorus.

Fig. 3 displays the elemental compositions of the surfaces of the TiO_2_ and Mn-TiO_2_ coatings, as determined by EDS. Ti, oxygen (O), Ca and P were detected in the TiO_2_ coating, while in the Mn-TiO_2_ coating, Mn was detected in addition to Ti, O, Ca and P. This indicated that Mn had been successfully incorporated into the coating. Table II summarizes the elemental compositions of the TiO_2_ and Mn-TiO_2_ coatings, and reveals that the Mn content was 2.41±0.08 wt% in the Mn-TiO_2_ coating. Following the Mn incorporation, the P content increased from 7.47±0.25 to 8.06±0.24 wt%, while the Ca content decreased from 7.09±0.21 to 6.04±0.19 wt%.

**Figure 4:**
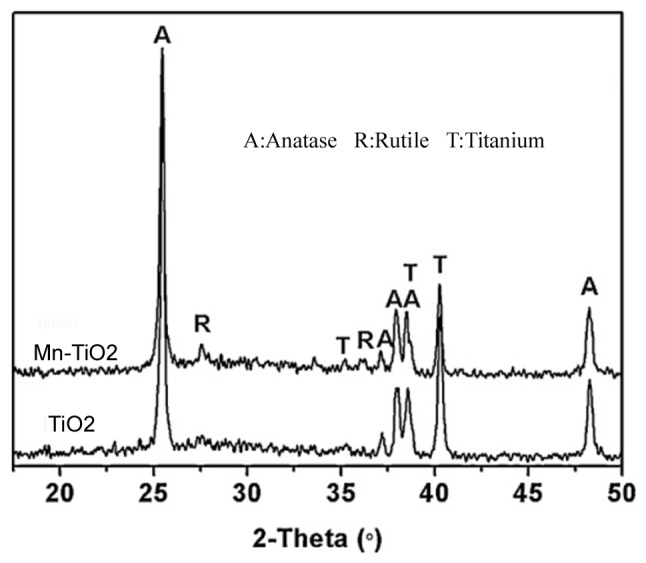
X-ray diffraction patterns of the titanium dioxide (TiO_2_) and manganese (Mn)-incorporated-TiO_2_ coatings.

The XRD patterns of the TiO_2_ and Mn-TiO_2_ coatings are displayed in Fig. 4. The two coatings primarily consisted of the anatase phase, while small peaks of the rutile phase were also detected in the XRD patterns of the Mn-incorporated sample. The incorporation of Mn only marginally altered the phase compositions of the TiO_2_ coating.

**Figure 5:**
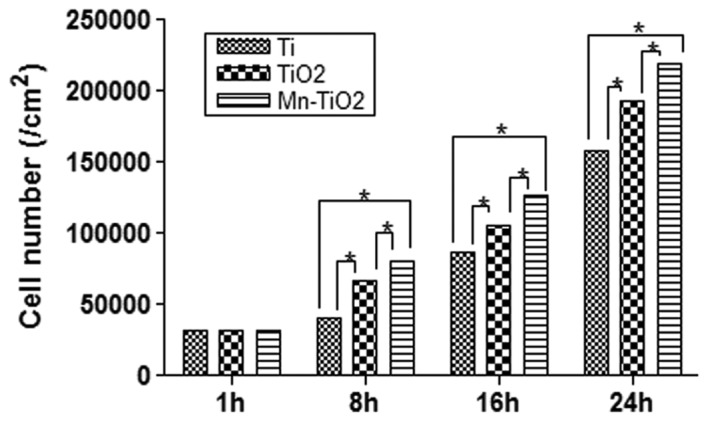
MG63 cell attachment onto titanium (Ti) plates, and titanium dioxide (TiO_2_) and manganese (Mn)-incorporated-TiO_2_ coatings. The results are expressed as cells/cm^2^, in functions of culture time. The osteoblasts were seeded at a density of 2×10^5^ cells/cm^2. *^P<0.05.

Fig. 5 displays the results of the MTT assay used to determine the attachment of the MG63 cells cultured on the Ti plates, and the TiO_2_ and Mn-TiO_2_ coatings at 1, 8, 16 and 24 h. During the initial 1 h of the incubation, the majority of the cells attached onto each of the substrates, and no significant differences were detected between them (P>0.05). However, the MTT assay indicated that there was a more rapid increase in cell attachment on the Mn-TiO_2_ coating, as compared with the Ti plates and TiO_2_ coating. At 8, 16 and 24 h, it was observed that the number of cells on the TiO_2_ coating was significantly greater than that on the Ti plates (P<0.05), but significantly less than that on the Mn-TiO_2_ coating (P<0.05). Therefore, the Mn-TiO_2_ coating appeared to provide a more favorable surface for the attachment of osteoblasts.

**Figure 6:**
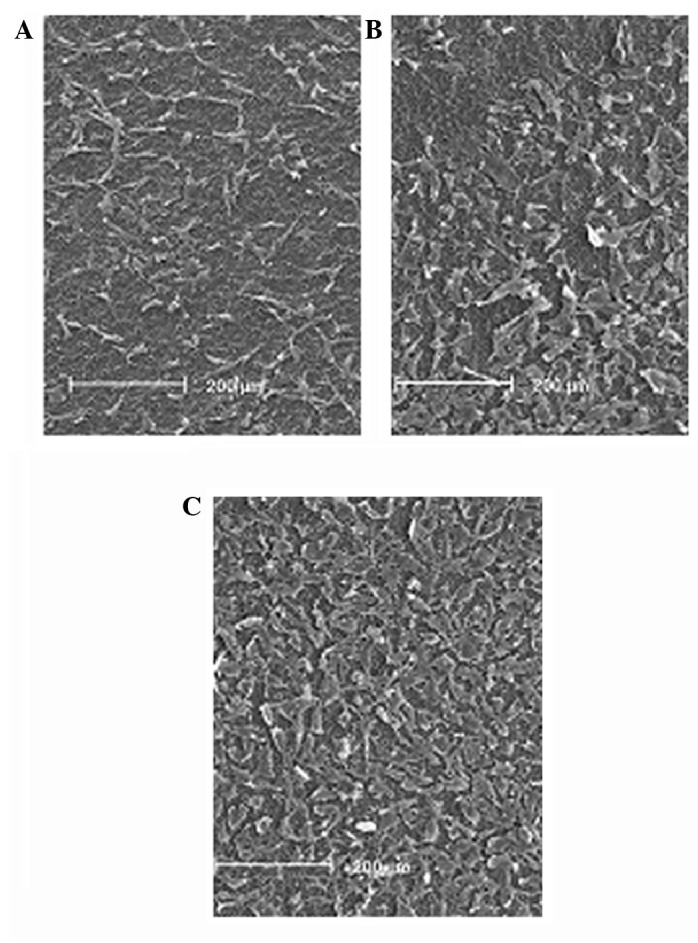
Scanning electron microscopy micrographs exhibiting the morphology of MG63 cells cultured for 16 h on (A) titanium plates, and with (B) titanium dioxide (TiO_2_) and (C) manganese (Mn)-incorporated-TiO_2_ coatings (bar=200 μm).

Fig. 6 reveals the osteoblastic morphology of the cells on the three substrates. The micrographs demonstrate that, following 16 h of culture on the Mn-TiO_2_ and TiO_2_ coatings, and the Ti plates, the MG63 cells exhibited different morphologies. In comparison with the cells seeded on the TiO_2_ coating and the Ti plates, those seeded on the Mn-TiO_2_ coating displayed a particularly spread-out morphology, with numerous connections to the surface.

**Figure 7:**
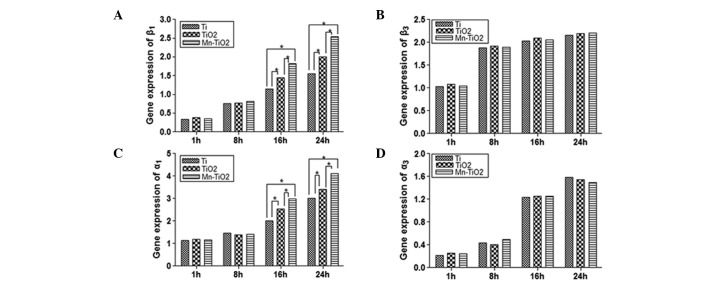
Gene expression of integrin (A) β_1_, (B) β_3_, (C) α_1_ and (D) α_3_ subunits. ^*^P<0.05. Ti, titanium; TiO_2_, titanium dioxide; Mn-TiO_2_, manganese-incorporated TiO_2_.

The expression of adhesion-specific genes, including integrins (subunits β_1_, β_3_, α_1_ and α_3_), FAK and ERK (ERK1 and −2), were measured at 1, 8, 16 and 24 h using qPCR. It was observed that the MG63 cells cultured on the Mn-TiO_2_ coating expressed higher levels of the mRNA of integrin (subunits β_1_ and α_1_), FAK and ERK2 compared with the cells seeded on the TiO_2_ coating and Ti plates. Integrins, as transmembrane heterodimeric receptors consisting of an α- and β-subunit, are important in mediating osteoblast adhesion onto biomaterials. Fig. 7 displays the integrin gene expression of MG63 cells cultured on Ti plates, and TiO_2_ and Mn-TiO_2_ coatings. The integrin β_1_ (Fig. 7A) and α_1_ (Fig. 7C) gene expression of the MG63 cells increased gradually with culture time, on each of the substrates. During the initial 8 h, the MG63 cells seeded on the three substrates demonstrated no significant differences in integrin β_1_ and α_1_ gene expression (P>0.05). When the MG63 cells were cultured on the substrates for ≥16 h, the gene expression of integrin β_1_ and α_1_ became significantly different, depending on the substrate. The Mn-TiO_2_ coating appeared to promote integrin β_1_ and α_1_ gene expression more effectively than the TiO_2_ coating and Ti plates, and resulted in the highest level of gene expression (P<0.05). There were no significant differences in the gene expression of integrin β_3_ or α_3_ between the cells seeded on the three substrates at any time; however, as the experiment progressed, the cells on each of the substrates demonstrated a marked upregulation in gene expression, which persisted throughout the course of the experiment (Figs. 7B and D).

**Figure 8:**
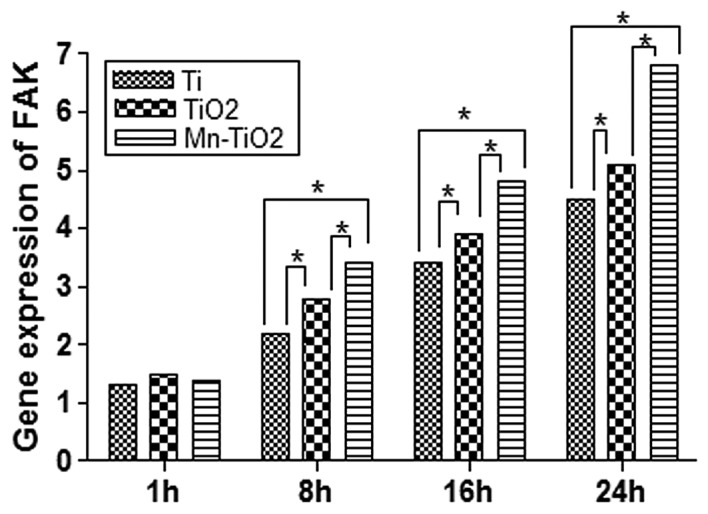
Gene expression of focal adhesion kinase (FAK). ^*^P<0.05. Ti, titanium; TiO_2_, titanium dioxide; Mn-TiO_2_, manganese-incorporated TiO_2_.

FAK, an integrin receptor, is important in the integrin-mediated signal transduction pathway, which mediates osteoblastic adhesion onto biomaterials. Fig. 8 demonstrates that at 1 h, the differences in FAK gene expression were not statistically significant (P>0.05). When the incubation time was ≥8 h, the FAK gene expression of the MG63 cells seeded on the TiO_2_ and Mn-TiO_2_ coatings was markedly upregulated. It was observed that the MG63 cells seeded on the Mn-TiO_2_ coating had a significantly higher level of FAK gene expression than those cultured on the TiO_2_ coating and Ti plates (P<0.05). The trend in FAK gene expression was similar to that of the cell attachment activity indicated in Fig. 5.

**Figure 9:**
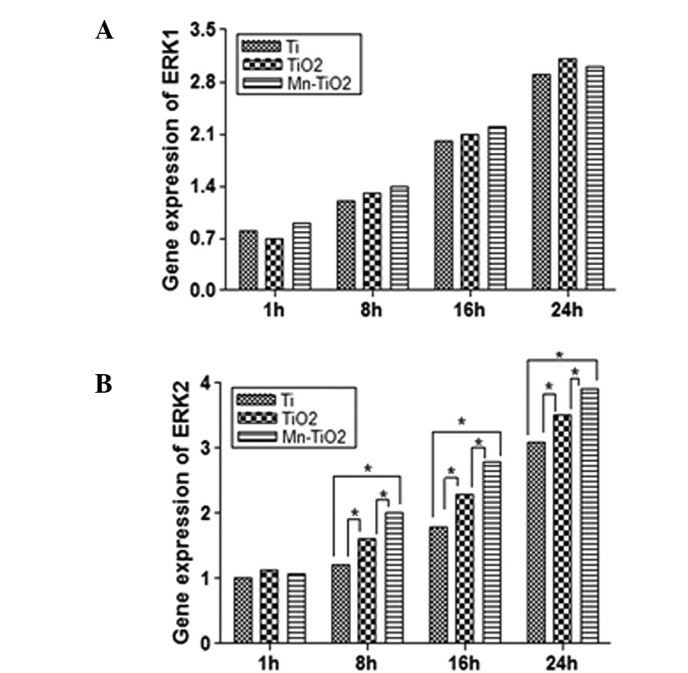
Gene expression of (A) extracellular regulated kinase (ERK)-1 and (B) ERK2. ^*^P<0.05. Ti, titanium; TiO_2_, titanium dioxide; Mn-TiO_2_, manganese-incorporated TiO_2_.

ERK (including ERK1 and −2), a member of the mitogen-activated protein kinase (MAPK) family, is essential in the regulation of osteoblastic adhesion, and may be stimulated as a result of FAK activation. The gene expression of ERK1 and −2 is displayed in Fig. 9. The results demonstrated that the ERK2 gene expression of the MG63 cells cultured on the Mn-TiO_2_ coating was upregulated at all time points, and became significantly greater than that of the cells on the TiO_2_ coating and Ti plates at 8, 16 and 24 h (Fig. 9B). With regard to the ERK1 gene expression, there were no significant differences between the three substrates at any of the time points, although the gene expression was upregulated continuously throughout the experiment (Fig. 9A).

## Discussion

The initial adhesion and spreading activities of osteoblasts characterize the first phase of cell-material interactions, and the quality of this stage influences the capacity of the cells to proliferate and differentiate on contact with the implant (21,22). Preclinical and clinical studies have demonstrated that osteoblastic adhesion on implant contact is significantly influenced by the surface properties of the implant, including the surface chemistry and topography (2,21,23). In the present study, we evaluated the adhesion of MG63 cells on three substrates (Ti plates, and TiO_2_ and Mn-TiO_2_ coatings), and investigated the relationship between the material of the substrate and the cell behavior.

As indicated by the MTT assay (Fig. 5), the attachment of a significantly greater number of cells to the Mn-TiO_2_ coating, compared with the TiO_2_ coating and the Ti plates, has certain implications for the long-term success of this material, due to the fact that the cell-surface integration is critical for the incorporation of the material into the new bone. Surface chemistry is an important factor affecting osteoblastic attachment to biomaterials (2,21). The Mn released from Mn-TiO_2_ coatings has been demonstrated to contribute to extracellular pH changes, which alter the structure of transmembrane proteins (9,10,11,14). This alteration of the transmembrane proteins has been revealed to facilitate the interaction and bonding of the transmembrane proteins with proteins adsorbed onto the Mn-TiO_2_ coatings from the culture medium, which promotes the attachment of MG63 cells onto the surface (7,10,11). In addition to surface chemistry, the attachment of osteoblasts is also affected by surface topography (21,24–27). Although a variety of studies (21,25,27–30) have revealed few consistent trends in the effects of surface topography on initial osteoblastic attachment, the most commonly observed trend has been that a porous structure is beneficial to cell attachment (15,17, 21,25,28,29). When the Mn-TiO_2_ coating is placed into the culture medium, the porous nanostructured surface facilitates the adsorption of proteins from the culture medium, by providing a larger contact area at the sample-medium interface. This promotes cell recruitment, and may be one of the reasons for the enhanced osteoblastic attachment on Mn-TiO_2_ coatings.

Osteoblastic spreading on the biomaterial is a process that is essential in establishing the biological properties of the cells (21,31,32), and may be significantly influenced by the surface chemistry of the biomaterial (2,21,33–35). It has been observed that incorporating Mn into a hyaluronan coating may promote the spreading of osteoblasts (8,9). In addition, Li *et al*(8) demonstrated that osteoblasts displayed a significantly flatter morphology on an Mn-containing coating. In the present study, we observed that following seeding on a Mn-TiO_2_ coating for 16 h, MG63 cells displayed a flatter morphology, and exhibited numerous connections to the surface, in comparison with cells on a TiO_2_ coating and Ti plates (as indicated in Fig. 6). The Mn released from the Mn-TiO_2_ coating may have bonded to oxygen, forming a Mn network structure on the surface of the coating, which was capable of holding elements of the proteins together in an organized fashion, thus contributing to the architecture of the connective tissue (6,8,10–12). It is possible that these proteins may have been adsorbed onto the Mn network structure, promoting enhanced osteoblastic spreading via interactions with the integrins on the MG63 cells and, in turn, triggering certain specific signals, which may have then had a stimulatory effect on the bone mineralization process. However, further investigation into the precise mechanism by which Mn affects osteoblastic spreading is required. The surface topography of the biomaterial is another factor that is important in osteoblastic spreading (21,24,26,27). Several studies have observed that bone cells spread and flattened with greater efficacy on nanostructured porous coatings, compared with rough coatings (21,36,37). However, contrary conclusions have been drawn in other studies (38,39), and, as a result, it has been difficult to establish a simple conclusion with regard to the correlation between the surface topography and osteoblastic spreading. In the present study, we demonstrated that the spreading of MG63 cells cultured on porous TiO_2_ and Mn-TiO_2_ coatings for 16 h was more pronounced than that on polished Ti plates (Fig. 6), suggesting that the spreading was promoted by the surrounding porous nanostructures.

When a biomaterial is placed in culture medium, proteins adsorb to its surface. This protein layer subsequently regulates the interaction of the biomaterial with the osteoblasts arriving from the surrounding tissue. Integrins are essential transmembrane molecules that are involved in the process of osteoblastic adhesion (1). It has been demonstrated that Mn increases the ligand-binding affinity of integrins, thus affecting cellular interactions with the extracellular matrix (ECM), and activating cell adhesion (10,11,13). The present study investigated the gene expression of the integrin subunits β_1_, β_3_, α_1_ and α_3_. We observed a significantly higher expression of the integrin β_1_ and α_1_ genes in the cells on the Mn-TiO_2_ coating compared with those on the TiO_2_ coating and Ti plates (Figs. 7A and C), following 16 h of culture, while no significant differences were observed with regard to the integrin β_3_ and α_3_ gene expression (Figs. 7B and D). The results indicated that the integrin β_1_ and α_1_ subunits may have been partially responsible for the enhanced osteoblastic adhesion on the Mn-TiO_2_ coating. However, a comparison of Fig. 5 and Fig. 7, revealed that although at 8 h a significantly greater number of MG63 cells were attached on the Mn-TiO_2_ coating than on the TiO_2_ coating or Ti plates, the levels of integrin β_1_ and α_1_ gene expression were approximately equal, irrespective of the substrate. This implied that alternative integrin subunits, in addition to β_1_ and α_1_, may have had an earlier effect on the process of MG63 cell attachment onto Mn-TiO_2_ coatings.

Following the binding of a ligand, integrins cluster together into focal contacts (1,40,41). This is an area of close contact between a cell and the ECM, and consists of additional cytoskeletal proteins, adapter molecules, and kinases (42). Subsequent to the clustering, cytoskeletal elements and signaling molecules are recruited and activated, in a process known as outside-in signaling (41,42). The signaling pathway inside the cell is complex, and involves the accumulation of several proteins, including FAK, Src, and cytoskeletal proteins (43). The activation of FAK initiates intracellular signal transduction cascades, including those involved in the MAPK effector cascades and the remodeling of the cytoskeleton. These effects, in turn, regulate cellular processes such as adhesion, growth, and differentiation (43). In the present study, it was observed that the FAK gene expression in the MG63 cells cultured on the Mn-TiO_2_ coating was significantly higher compared with that of the cells cultured on a TiO_2_ coating or Ti plates at 8, 16, and 24 h (Fig. 8). This revealed a similar trend to that of the gene expression of the integrin β_1_ and α_1_ subunits, indicating that these subunits may have been involved in stimulating FAK activation at 16 and 24 h. It is possible that alternative integrin subunits may have contributed to the activation of FAK, leading to the higher expression of FAK in the cells on the Mn-TiO_2_ coating at 8 h.

Integrin and FAK molecules may provide a platform for intracellular signaling; however, they are not able to exhibit intrinsic enzymatic activity in their cytoplasmic domains. Downstream signaling, following integrin binding, is regulated by non-receptor tyrosine kinases (43), and one such pathway is the ERK/MAPK signaling cascade (44). Several studies have demonstrated that integrin engagement and FAK activation stimulated ERK expression (43,44). In the current study, we observed that the gene expression of ERK2 (Fig. 9B) was similar to that of the integrin β_1_, α_1_ subunits (Fig. 7A and C) and FAK (Fig. 8), which implied that integrin β_1_ and α_1_-mediated activation of FAK may have been responsible for the enhanced ERK2 expression on the Mn-TiO_2_ coating at 16 and 24 h. The increased ERK2 gene expression at 8 h on the Mn-TiO_2_ coating may have been induced by alternative integrin subunits, in addition to β_1_ and α_1_.

In conclusion, the present study prepared a porous and nanostructured Mn-TiO_2_ coating by PEO, using a novel Mn-containing electrolyte, which successfully incorporated Mn into the coating. The microstructure, Ra and phase composition of the TiO_2_ coating were not altered following the incorporation of Mn; however, the Mn-incorporated TiO_2_ coating was demonstrated to exhibit biological activity in promoting the adhesion of MG63 cells. Moreover, the present study indicated that Mn-TiO_2_ coatings may modulate osteoblastic proliferation and differentiation, a process regulated by the ERK/MAPK signaling pathway, through integrin-FAK mediated cellular adhesion. There are limitations to using an evaluation of initial cell adhesion as an end point for a screening assay of potential material surfaces; however, further studies involving *in vitro* cellular calcification and mineralization assays, along with *in vivo* histological observations are currently in progress.

## Figures and Tables

**Table I:** Primer pairs used in the present study.

Target	Primer sequence
Integrin α_1_	F: 5′-TCGCTCCGTGGCCTTGTGGAA-3′
	R: 5′-CCCATTTCAGTAACCACGCCC-3′
Integrin α_3_	F: 5′-TGGGTCATAGACCGGTATAC-3′
	R: 5′-ATCCACTCATAGCAAACAGT-3′
Integrin β_3_	F: 5′-TGCTGACGGGAGGAACGGTA-3′
	R: 5′-CGGGATCAGATGTGTCTGGG-3′
Integrin β_1_	F: 5′-GTCTTTGCGTAGGCTTACTT-3′
	R: 5′-ACCCGTGGGTACGATGCATC-3′
FAK	F: 5′-GCGAGAGGTGTGGTAATATCAGGTGAA-3′
	R: 5′-ACACGTATTCACTGTTCAACTATTGAC-3′
ERK1	F: 5′-ACACACGCCCAAACCATG-3′
	R: 5′-TCCACTCGCGCATTCGTA-3′
ERK2	F: 5′-TTTACCATAGGACTCAACCT-3′
	R: 5′-GGGCATGGTGTCCTGCAGAA-3′
GAPDH	F: 5′-CGCGTCGCGCTCGATGTCAC-3′
	R: 5′-GGTAAGTGACATGCTGAGTT-3′

F, forward; R, reverse; FAK, focal adhesion kinase; ERK, extracellular regulated kinase; GAPDH, glyceraldehyde-3-phosphate dehydrogenase.

**Table II:** Elemental compositions of the TiO_2_ and Mn-TiO_2_ coatings, detected by EDS.

	Elemental composition (weight %)				
	
Samples	Ca	P	Ti	O	Mn
TiO_2_	7.09±0.21	7.47±0.25	43.55±0.44	41.89±0.42	-
Mn-TiO_2_	6.04±0.19	8.06±0.24	37.31±0.36	46.18±0.49	2.41±0.08

Mn-TiO_2_, manganese-incorporated titanium dioxide; EDS, energy dispersive spectrometry; Ca, calcium; P, phosphorus; Ti, titanium; O, oxygen; Mn, manganese.
